# Insurance Status and Biological and Psychosocial Determinants of Cardiometabolic Risk Among Mexican-Origin U.S. Hispanic/Latino Adults with Type 2 Diabetes

**DOI:** 10.1089/heq.2019.0119

**Published:** 2020-05-04

**Authors:** Namino M. Glantz, Jessikah M. Morales, Wendy C. Bevier, Arianna Larez, Charis B. Hoppe, Ian Duncan, Andrew Mackenzie, David Kerr

**Affiliations:** ^1^ Sansum Diabetes Research Institute, Santa Barbara, California, USA.; ^2^ Department of Statistics and Applied Probability, University of California Santa Barbara, Santa Barbara, California, USA.; ^3^ Santa Barbara Actuaries, Santa Barbara, California, USA.

**Keywords:** Hispanic, Latino, diabetes, cardiometabolic risk, health insurance

## Abstract

**Purpose:** Hispanics/Latinos in the United States bear higher burden of type 2 diabetes (T2D) and associated complications compared with the general population. Health insurance coverage is also lower in this population. We examined the association of health insurance with biological and psychosocial determinants of cardiometabolic risk among U.S. Mexican-origin Hispanic/Latino adults with T2D.

**Methods:** Participants were self-reported Hispanic/Latino adults with T2D diagnosis. Trained bilingual community health workers collected cross-sectional information on biological and psychosocial factors using clinical examinations, laboratory tests, validated questionnaires, and wearable activity monitors.

**Results:** One hundred and seven Hispanic/Latino adults (54±12 years, 65% female, 36% prescribed insulin, 60% uninsured) with T2D were enrolled. While 93% had low language-based acculturation, 88% had high health literacy in Spanish. Forty percent were food insecure and 47% expressed at least one social need. Overall, 35% had an HbA_1c_ <7.0% (indicating good control) and 31% had an HbA_1c_ >9.0%. Sixty-three percent had blood pressure within target (<130/80 mmHg), and overall participants were moderately physically active. However, 53% were obese (body mass index ≥30 kg/m^2^) and 76% had a waist measurement defined as high risk (>88 cm for women and >102 cm for men). Participants without health insurance were younger (51.9±10.4 vs. 58.8±10.5 years mean±standard deviation, *p*=0.0008) but had higher HbA_1c_ (8.4±2.2% vs. 7.6±1.6, *p*=0.031) and fasting glucose (184.9±86.5 vs. 148.6±61.2 mg/dl, *p*=0.008) levels.

**Conclusions:** Health insurance status appears to influence achieved glycemic control for U.S. Hispanic/Latino adults with T2D. However, various psychosocial factors potentially influencing cardiometabolic risk independently of health insurance status may also be implicated in the inequitable burden of T2D. ClinicalTrials.gov Identifier: NCT03736486.

## Introduction

In the United States, racial and ethnic minority groups are disproportionally impacted by diabetes, especially type 2 diabetes (T2D). The prevalence of diagnosed and undiagnosed T2D among Mexican-origin Hispanic/Latino adults is nearly double that in non-Hispanic white adults.^1^ Furthermore, rates of diabetes-related complications, including premature death, acute stroke, and end-stage renal disease, are higher among Hispanic/Latino adults than non-Hispanic whites.^2^ Additional concerns are worsening rates of detection of previously undiagnosed T2D among Hispanic/Latino adults versus non-Hispanic white, very high income, and older adults.^3^

Among established clinical drivers of excess T2D burden on the U.S. Hispanic/Latino population are delays in diabetes diagnosis,^4^ suboptimal blood glucose control, and low rates and frequency of self-monitoring and screening for complications.^5–7^ Beyond biological factors, such as HbA_1c_, sociocultural influences are also important determinants of T2D risk. These include ethnicity (e.g., greater T2D burden in Mexican Americans than Cuban Americans), acculturation, education, economic status, and residence.^8,9^ Another potentially relevant factor is access to health insurance. In the United States, lack of health insurance influences access to and participation in treatment plans for diabetes diagnosis, self-management, and use of timely and appropriate therapies.^10^ In turn, health insurance status may impact both clinical determinants (e.g., HbA_1c_) and psychosocial determinants (e.g., stress) of health, and influence ability to reduce personal diabetes risk.

Nationally, U.S. Hispanic/Latino individuals are disproportionately affected by lack of health insurance.^11^ Furthermore, racial and ethnic disparities exist in the burden and cost of diabetes care even for those with health insurance.^12^ Among Hispanic/Latino individuals with diabetes, those lacking health insurance have higher rates of microvascular complications.^13^ Thus, expanding health insurance access has been suggested as having potential to eliminate health disparities among Hispanic/Latino populations. The aim of this study was to determine whether there is a relationship between health insurance coverage and biological and psychosocial determinants of health among Hispanic/Latino adults with T2D in a California Central Coast community in which Hispanic/Latinos comprise 45% of the population, most of Mexican origin.^14^

## Methods

The study was approved by an independent review board (Quorum 32669/1). Eligible participants were of self-reported Hispanic/Latino heritage, aged ≥18 years with a ≥1 year T2D diagnosis. Individuals were excluded if they self-reported a diagnosis of severe cardiovascular disease within the prior 6 months limiting their ability to attend study visits, a likelihood of <2 years life expectancy, and/or participation in other trials involving medication or device/s within the prior month.

### Recruitment and consent

Participants were recruited through existing Sansum Diabetes Research Institute (SDRI) and community outreach and service programs as well as a local federally qualified health center (FQHC) that provides safety net care to Hispanic/Latino families. Individuals amenable to sharing their contact information were referred to trained bilingual community health workers known as Especialistas, who provided IRB-approved verbal and printed information about study aims and requirements. Especialistas then facilitated informed consent with those interested in participating.

All study materials were available in English and Spanish, and activities were conducted in the participant-preferred language/s. Validated questionnaires were approved for translation by the original authors and translated through certified translation services. Especialistas reviewed methods and materials for cultural congruence.

### Participant activities

Once consented, participants completed a study visit series for data collection. Biological data were captured through a clinical examination by a medically qualified provider at the FQHC and a blood draw at a partnering laboratory. At SDRI, Especialistas collected psychosocial and behavioral data by applying validated questionnaires and wearable activity monitors. Participants completed visit activities in the following order:

(1) Consent, enrollment, questionnaires
(2) Laboratory analyses
(3) Physical examinations
(4) Wearable activity monitoring, questionnaires.
(5) Conclusion

On average, participants completed visits pertinent here (1–4) within 6 months of enrollment (minimum 3, maximum 11, median 4 months).

At Visit 1, after consent, Especialistas collected sociodemographic information, including health insurance status, household size, educational attainment, and employment, using an SDRI-developed Introductory Interview (II). Participants next answered brief open-ended questions about diabetes in their family, their perceptions of what diabetes is, why it is common in Hispanic/Latino families, and how to prevent and address it. Participants then completed three questionnaires validated in Hispanic/Latino populations. The 6-item U.S. Household Food Security Survey Module short form (HFSSM) produces a score of high, marginal, low, or very low food security.^15^ The Social Needs Screening (SNS) is a 9-item questionnaire with “yes” or “no” answers regarding common social determinants of health across five domains: utilities, housing, childcare, health care, and domestic violence.^16–18^ The brief Perceived Self-Assessment of Diabetes Management (PSADM) probes support, dietary behavior, learning style, cultural and religious practices, and handling stress, using 10 multiple-choice and short-answer questions.^19^

At Visit 2, participants were asked to arrive fasting unless insulin treated. A blood draw was collected for HbA_1c_, complete metabolic and lipid panel, full blood count, thyroid function, and insulin levels (for noninsulin users). Laboratory analysis was completed by a commercial laboratory in partnership with the FQHC. Results were accessed through secure patient electronic medical records. Homeostatic model assessment for insulin resistance (HOMA-IR) was calculated as HOMA-IR score=fasting insulin (μIU/mL or mU/L)×fasting glucose (mg/dL)/405.^20^

At Visit 3, an FQHC provider recorded medical history and medications; performed a physical examination (including height, weight, and waist circumference); and screened for common diabetes-related complications (e.g., eye, kidney, nerve, and circulation damage). Relative fat mass (RFM) was calculated by the Woolcott and Bergman 2018 method.^21^ The 9-item Likert scale Patient Health Questionnaire-9 (PHQ-9) was applied to screen for depression.^22^

Subsequently, at Visit 4, participants met with Especialistas to complete additional psychosocial questionnaires validated in Hispanic/Latino populations. The Oviedo Sleep Questionnaire (OSQ) uses 11 Likert scale items to assess subjective sleep quality, insomnia, hypersomnia, and use of sleep aids.^23,24^ The Perceived Stress Scale (PSS-4) evaluates frequency of stress over the previous month using 4-item Likert scale; scoring yields low or high stress ratings.^25^ The Perceived Ethnic Discrimination Questionnaire-Community Version (PEDQ-CV) consists of 17 Likert-scaled questions to assess frequency of discrimination based on ethnicity to yield subscale scores for exclusion/rejection, stigmatization/devaluation, threat/aggression, and discrimination at work or school, and lifetime exposure to discrimination.^26^ The 4-item Likert-scaled Brief Acculturation Scale for Hispanics (BASH) probes comfort with English and Spanish language use as a proxy for low and high acculturation.^27^ Finally, the Short Assessment of Health Literacy (SAHL) is an 18-item exercise to assess ability to read and understand common medical terms in the participant's preferred language (predominantly Spanish).^28^ Participants were shown flashcards that contain three words, and were asked to identify the correct synonym to the first word on the card; scoring indicated low or high health literacy.

Participants were then provided with two wearable physical activity monitors: an ActiGraph wGT3X-BT (ActiGraph, LLC, Pensacola, FL) worn around the waist on the dominant hip, and a Fitbit Charge 2^™^ (Fitbit, Inc., San Francisco, CA) worn on the nondominant wrist. Especialistas trained participants on wearable device use and care, instructing them to wear the devices for at least 6 days and nights, removing them only for bathing or swimming. After a week, participants returned both devices to SDRI.

Finally, Visit 5, dedicated to closing out the visit series, took place at least 1 week after Visit 4. In this visit, Especialistas applied SDRI-developed questionnaires to capture information on participant experience in the study.

Perceptions of the meaning of disease are influential in health outcomes, with favorable illness perception associated with better health outcomes, and unfavorable perception associated with worse outcomes.^29,30^ To explore this possibility, we went beyond biological descriptors and quantitative psychosocial metrics (e.g., achieved HbA_1c_, weight, insulin use, and depression) to explore qualitative perceptions of diabetes. Specifically, two independent bilingual raters conducted thematic analysis of answers to the question, “In your own words, what is diabetes?” This yielded five categories: controllable, sugar, body malfunction, illness, doom/death.^31^ Doom/death was a prominent theme in participant definitions of diabetes, with participants using words such as bad, sad, serious, degenerative, incurable, torturous, lifelong, and deadly.

### Statistical analyses

Means and standard deviation (SD) were calculated for all variables except steps per weekday and weekend. The Shapiro–Wilk test showed that accelerometer values were not normally distributed; therefore, median and range were calculated for steps per day. Between-group comparisons were made using a Welch's *t*-test. Additional comparisons of proportions were made using the chi-squared test. Significance thresholds were set at the 95% level. Differences for steps per day for weekdays and weekends were compared using Mann–Whitney U tests between insured versus uninsured.

To probe possible generalizability and transferability of methods and findings from this pilot sample to larger populations, results were compared with those of a large established cohort.^32,33^ The Hispanic Community Health Study/Study of Latinos (HCHS/SOL) is a community-based cohort study of 16,415 self-identified Hispanic/Latino adults, of which 2148 individuals self-reported a diagnosis of diabetes, and of whom 856 self-identified as of Mexican heritage.

## Results

Between September 2017 and August 2018, 107 Hispanic/Latino adults with T2D (95% of Mexican heritage, 67% female) provided written informed consent and enrolled in the study. Participant activities culminated in October 2018. Nearly all participants (98%) opted to use Spanish (over English) study materials. All variables reported in this study were collected (in Visits 1–4) from 50% of participants within 3 months of enrollment, and from 90% of participants within 6 months of enrollment (median 4 months).

### Insurance status

Of 101 participants reporting their current health insurance status, 61 (60%) had no insurance and 40 (40%) had insurance at study initiation. Among the 40 participants who were insured upon enrollment, the majority (31) were covered by a Medi-Cal plan.

### Biological and behavioral determinants and health insurance status

At enrollment, 36% of participants had been prescribed insulin, 46% a statin, and 31% blood pressure lowering medication. Thirty-five percent had an HbA_1c_ <7.0% and 31% had an HbA1_c_ >9.0%. More than half (63%) of participants had blood pressure <130/80 mmHg. Based on body mass index (BMI) criteria, 12% of subjects had a normal value (18.5–24.9 kg/m^2^), whereas 53% were in the obese range (BMI ≥30 kg/m^2^). Three-quarters (76%) of overall participants had a waist measurement defined as high risk (>88 cm for women and >102 cm for men), with an average measurement of 103.8±13.5 cm (mean±SD) among women and 105.1±12.6 cm among men.^34^

Table 1 displays participant biometrics stratified by insurance status. Of these 12 different biological determinants, 4 significantly differed by insurance status:

▪ Uninsured participants were younger than insured participants.
▪ Uninsured participants had higher HbA_1c_ than insured participants.
▪ Uninsured participants had higher fasting glucose than insured participants.
▪ Uninsured participants had a smaller waist circumference than insured participants.

**Table 1.** Biometric Descriptors by Health Insurance Status (Mean±Standard Deviation)

Biometric	Uninsured	Insured	p
Age (in years)	51.9±10.4	58.8±10.5	0.0008
HbA_1c_ (%)	8.4±2.2	7.6±1.6	0.0307
Fasting glucose (mg/dL)	184.9±86.5	148.6±61.2	0.0079
Waist circumference (cm)	101.9±10.5	108.7±16.3	0.0223
Waist circumference (female)	102.1±11.8	106.9±16.0	NS
Waist circumference (male)	101.7±7.9	111.7±17.1	NS
Systolic blood pressure (mmHg)	123.1±17.1	128.4±15.5	NS
Body mass index (kg/m^2^)	30.8±6.0	32.8±6.4	NS
Weight (kg)	76.9±15.7	83.4±19.7	NS
Relative fat mass	40.2±7.7	41.8±8.1	NS
HOMA-IR	4.8±2.2	4.6±2.2	NS
LDL cholesterol (mg/dL)	97.5±35.1	101.3±28.3	NS
Total cholesterol (mg/dL)	183.5±57.3	179.4±39.6	NS
Triglycerides (mg/dL)	170.7±111.3	166.7±112.7	NS

HOMA-IR, homeostatic model assessment for insulin resistance; LDL, low-density lipoprotein; NS, not significant.

Overall, ActiGraph and Fitbit showed moderate physical activity levels (Table 2). However, the average step count per day reported by ActiGraph was significantly lower compared with Fitbit (median [range] 7145.8 [1953.3–18,668.3] vs. 8674.7 [1946.7–24,544.3]; [*p*<0.05]). There was a significant difference between uninsured and insured for average steps per day and average steps per weekday measured by ActiGraph, with the uninsured showing more activity than the insured. No significant differences were seen for average steps per weekend, or for steps per day, weekday, or weekend measured by Fitbit.

**Table 2.** Average Step Counts per Day Assessed by ActiGraph and Fitbit for Weekdays, Weekends, and All Days of the Week by Health Insurance Status

Device and day type	Median (range)		p
	Uninsured	Insured	
ActiGraph measured average steps/weekday	7945.6 (3880.5–19,481.3)	6193.0 (1840.3–18,423.6)	<0.05
ActiGraph measured average steps/weekend	7083.5 (1543.0–18,375.0)	5256.0 (1560.5–19,892.0)	NS
ActiGraph measured average steps per day	7368.1 (3734.3–15,310.6)	5770.2 (1953.3–18,668.3)	<0.05
Fitbit measured average steps/weekday	8654.0 (1174.2–24,561.3)	9828.4 (1171.8–21,223.0)	NS
Fitbit measured average steps/weekend	7675.5 (3172.5–20,855.0)	6674.5 (1775.5–18,235.0)	NS
Fitbit measured average steps per day	8644.5 (1946.7–24,544.3)	8873.3 (2059.5–18,229.7)	NS

Significance was measured using Mann–Whitney U tests.

### Psychosocial determinants and health insurance status

In general, participants were <65 years of age (84%), with most living in 3+ person households (78%). Forty percent reported food insecurity. More than half had less than a high school education (55%) and were unemployed (57%). Nearly half (47%) expressed at least one social need (utilities, housing, childcare, health care, and/or domestic violence). Nearly all (94%) had immigrated to the United States, primarily from Mexico. Most had low language-based acculturation (93%), but high health literacy in Spanish (88%). The majority indicated sleep satisfaction (60%), no depression (72%), and low stress (78%).

Table 3 displays psychosocial determinants stratified by insurance status. Of these 12 psychosocial factors, 3 significantly differed by insurance status:

▪ Uninsured participants were younger than insured participants.
▪ Uninsured participants lived in larger households than insured participants.
▪ Uninsured participants were more food secure than insured participants.

**Table 3.** Psychosocial Determinants of Health by Health Insurance Status

Determinant	Uninsured %	Insured %	χ^2^
Age (years)			
<65	90.1	75.0	4.17^a^
≥65	9.8	25.0	
Household size			
1–2 Person household	13.1	37.5	8.17^a^
3+ Person household	86.9	62.5	
Food security			
Low food security	31.1	55.0	5.70^a^
High food security	68.9	45.0	
Education			
Less than high school	59.6	57.9	NS
High school or more	40.4	42.1	
Employment status			
Unemployed	57.4	57.5	NS
Employed	42.6	42.5	
Social needs			
0 Social needs	47.5	57.5	NS
1 or More social needs	52.5	42.5	
Immigrated to the United States			NS
No	4.9	7.5	
Yes	95.1	92.5	
Acculturation			
Low acculturation	92.7	94.4	NS
High acculturation	7.3	5.6	
Health literacy			
Low health literacy	14.6	8.3	NS
High health literacy	85.4	91.7	
Sleep satisfaction			
Very satisfied/satisfied	60.0	58.3	NS
Dissatisfied	40.0	41.7	
Depression			
No depression	72.0	72.7	NS
Depression	28.0	27.3	
Perceived stress			
Low stress	72.7	86.1	NS
High stress	27.3	13.9	

^a^ *p*<0.05 comparing uninsured with insured.

When stratified by insurance status, there was no significant difference between uninsured and insured participant likelihood to define diabetes as doom/death.

### Comparison with HCHS/SOL

To probe generalizability and transferability from this pilot sample to larger populations, we compared our results with those of the large established cohort of Hispanic/Latino adults with T2D from the HCHS/SOL.^33^ Comparisons of biological determinants between our study population and the 856 total HCHS/SOL participants of Mexican heritage with T2D are shown in Table 4. In general, in terms of age, HbA_1c_, waist circumference, blood pressure, BMI, and low-density lipoprotein cholesterol, the two cohorts appear to be similar.

**Table 4.** Comparison of Biological Determinants in Current Cohort with the Hispanic Community Health Study/Study of Latinos Cohort

Determinant	Current cohort (n=107)	HCHS/SOL cohort: Mexican adults with T2D (n=856)
	Percentage	Percentage
Age (years)		
22–39	10.3	19.0
40–59	58.9	51.9
60–80	30.8	29.1
HbA_1c_ <7.0%	35.6	41.6
HbA_1c_ ≥9.0%	30.7	31.4
High risk waist circumference, >88 cm women, >102 cm men	76.0	70.0
Blood pressure >130/80 mmHg	41.0	41.0
Body mass index, kg/m^2^		
18.5–24.9	11.8	10.4
25.0–29.9	34.4	35.7
30.0–34.9	30.1	30.0
≥35.0	22.6	23.9
LDL-cholesterol >100 mg/dL	45.0	62.0

HCHS/SOL, Hispanic Community Health Study/Study of Latinos; T2D, type 2 diabetes.

The HCHS/SOL included few psychosocial variables similar to this study. Two psychosocial areas in which comparisons could be made are depression and social needs. In our study of Hispanic/Latinos predominantly of Mexican origin, the rate of depression was 28%, which is similar to the rate in the HCHS/SOL cohort of Hispanic/Latino adults of any origin (27%), but higher than the rate among HCHS/SOL participants of Mexican origin (22%).^35^ In terms of social needs, in our study, the most prominent need was “could not see a physician because of cost” (13%). Among the Mexican participants with diabetes in the HCHS/SOL, nearly 15% had needed health care in the past 12 months but could not get it.^33^

## Discussion

In the United States, the Hispanic/Latino population is the fastest growing ethnic minority group and is known to have an excess burden of diabetes and associated complications compared with the general population.^1^ Health insurance coverage has been cited as a factor in diabetes diagnosis, self-management, use of timely and appropriate therapies, and, ultimately, clinical health outcomes for the general population.^10^ In this study, we examined the association of health insurance status with biological and psychosocial determinants of health for U.S. Hispanic/Latino adults, predominantly of Mexican origin, with an established diagnosis of T2D.

Of 12 biological determinants of health assessed in this study, 4 differed significantly by insurance status: age, HbA_1c_, fasting glucose, and waist circumference. Notably, from a purely glycemic perspective, having health insurance was associated with better glycemic control. However, both insured and uninsured groups had unfavorable cardiovascular risk profiles with high rates of hyperlipidemia, obesity, insulin resistance, and excess levels of high-risk waist measurements, although average waist circumference was significantly higher among the insured group. This clinical profile indicates a population in poor health and at risk for complications stemming from inadequate diabetes management, regardless of insurance status.

Accelerometer and consumer device measured physical activity assessment suggested fairly high levels of physical activity in this study population, regardless of insurance status and consistent with previous data.^36^ Overall, insured and uninsured participants alike came close to or met the recommended 10,000 daily steps.^37^ Furthermore, we detected consistent differences between step counts recorded by both devices during the week compared with weekends.^38^ ActiGraph captured significantly higher average steps per day and average steps per weekday among uninsured participants than insured participants. Both physical activity rates and insurance status may be independently associated with occupation.

Two of the 12 psychosocial determinants of health assessed in this study were associated with having health insurance: living in a smaller household and food insecurity. It may be that people must choose to invest in either insurance or food, and/or that people with low income without health insurance qualify for food benefits (e.g., Supplemental Nutrition Assistance Program), whereas employed insured people earn too much to qualify for benefits yet not enough to purchase adequate food. Regardless of insurance status, we found high rates of health literacy and sleep satisfaction, and low rates of depression and stress, which may contrast with the larger U.S. population with T2D, and may be potentially advantageous to this Hispanic/Latino, predominantly Mexican-origin subpopulation.

Finally, we compared the overall study population with a larger cohort of Hispanic/Latino adults with T2D cohort published previously.^30^ We confirmed various biological similarities as well as indication of various psychosocial similarities between the two samples.

## Limitations

Untangling the association between and potential influence of T2D status on access to health insurance—and conversely the impact of insurance status on diabetes outcomes—is challenging. The relatively small sample size, gender division of the sample, and geography from which the sample was obtained limit interpretation of the findings. In addition, the heritage of participants was overwhelmingly Mexican and thus findings may not be applicable to non-Mexican-origin groups comprising the wider Hispanic/Latino population. Data were collected for 4–6 months, during which time insurance status, biological, behavioral, and psychosocial variables may have changed. There are also probable biases between the risk profiles of the insured compared with uninsured populations in this study. It is possible that individuals without insurance might be at higher risk because they do not have sufficient access to preventative and maintenance treatments and medical care. However, it is also possible that Hispanic/Latinos with health insurance might be at higher risk because of more health concerns and thus be more likely to purchase health insurance than those with fewer health issues that require maintenance treatment. There is some evidence to support the latter scenario, as the insured group was older and had a larger waist measurement, as well as a tendency for higher systolic blood pressure and BMI, compared with the uninsured group.

## Conclusions and Health Equity Implications

In conclusion, contrary to the prevalent and often evidence-based assertion that health insurance is an impactful determinant of diabetes outcomes, we did not find many associations between health insurance and the biological and psychosocial metrics assessed in this sample of Hispanic/Latino adults with T2D, most of Mexican origin. This underscores the need to assess the relationship between myriad biological and psychosocial factors and T2D in U.S. subpopulations.^39^ The similarities between characteristics in our cohort and the larger nationwide HCHS/SOL cohort suggest that our findings in this small localized community in the California Central Coast may be representative of Hispanic/Latino populations nationwide. The contrast between both Hispanic/Latino cohorts and the general population evidences the need to explore in subpopulations the role of health insurance alongside biological and other psychosocial determinants, such as food security and social support, known to impact diabetes self-management in underserved populations with T2D.^40–42^

## Abbreviations Used

BMI: body mass index
FQHC: federally qualified health center
HCHS/SOL: Hispanic Community Health Study/Study of Latinos
HOMA-IR: homeostatic model assessment for insulin resistance
SD: standard deviation
SDRI: Sansum Diabetes Research Institute
T2D: type 2 diabetes
